# Approaches for extracting daily dosage from free-text prescription signatures in heart failure with reduced ejection fraction: a comparative study

**DOI:** 10.1093/jamiaopen/ooae153

**Published:** 2025-01-03

**Authors:** Theodorus S Haaker, Joshua S Choi, Claude J Nanjo, Phillip B Warner, Ameen Abu-Hanna, Kensaku Kawamoto

**Affiliations:** Department of Biomedical Informatics, University of Utah, Salt Lake City, UT 84108, United States; Department of Medical Informatics, University of Amsterdam, 1105 AZ Amsterdam, The Netherlands; Department of Biomedical Informatics, University of Utah, Salt Lake City, UT 84108, United States; Department of Internal Medicine, University of Utah Health, Salt Lake City, UT 84112, United States; Department of Biomedical Informatics, University of Utah, Salt Lake City, UT 84108, United States; Department of Biomedical Informatics, University of Utah, Salt Lake City, UT 84108, United States; Department of Medical Informatics, University of Amsterdam, 1105 AZ Amsterdam, The Netherlands; Department of Biomedical Informatics, University of Utah, Salt Lake City, UT 84108, United States

**Keywords:** daily dosage extraction, clinical information extraction, structured medication order, electronic prescribing, daily dosage

## Abstract

**Objective:**

To compare various methods for extracting daily dosage information from prescription signatures (sigs) and identify the best performers.

**Materials and Methods:**

In this study, 5 daily dosage extraction methods were identified. Parsigs, RxSig, Sig2db, a large language model (LLM), and a bidirectional long short-term memory (BiLSTM) model were selected. The methods were analyzed with regard to positive predictive value (PPV), sensitivity, F1-score, cost to compute, and time to finish on a sig dataset in the context of heart failure with reduced ejection fraction.

**Results:**

The dataset consisted of 29 896 free-text sigs, which were split into training and validation sets of 70% and 30%, respectively. The BiLSTM model scored lowest with an F1-score of 0.71. The LLM GPT-4o and regular expression-based RxSig achieved the highest F1-scores with 0.98 and 0.95, respectively. The LLM outperformed RxSig in sensitivity. RxSig outperformed the LLM in PPV. Additionally, RxSig had a lower run time and no costs compared to a cost of 25 dollars.

**Discussion:**

In practical usage, it would be preferable for an algorithm to score high on PPV and F1-score, to reduce false positive assertions of daily dosage. Additionally, long running times and high costs are not scalable for larger datasets. Thus, RxSig is likely the most scalable approach. Further research is needed to investigate the generalizability of the findings.

**Conclusion:**

This study demonstrates that both the LLM and RxSig models excel in daily dose extraction from free-text sigs, with the RxSig model appearing to be the more scalable approach.

## Introduction

Medications are a central part of clinical care.^1^ When medications are ordered, a prescription signature (sig) specifies how the patient should take the medication (eg, “take one tablet twice daily”). Of the attributes extractable from a sig, one of the most important attributes is daily dosage. This information is important as it helps determine whether patients are taking safe and effective dosages. In the context of our study of heart failure with reduced ejection fraction (HFrEF), it is critical that the daily dosages of medications shown to reduce mortality in HFrEF are titrated to their recommended daily doses.^2^

Ideally, a medication order would capture the elements of its sig in a structured format, such as dosage = 1, dosage unit = tablet, and frequency = twice a day. However, clinicians can choose to skip the structured entry fields and enter the sig in a free-text format. Due to variability in free-text sigs, automatically interpreting medication orders is challenging, especially in critical use cases such as determining whether a patient is receiving the recommended therapeutic daily dosage of a medication.^3^

The task of extracting these structured elements from unstructured text becomes essential, prompting the need for information extraction techniques.^4^ Techniques employed for extracting information from clinical text include the use of regular expressions, part-of-speech tagging using Unified Medical Language System (UMLS) concepts, and machine learning methods including large language models (LLMs).^4–6^ However, an important gap in the literature is that these methods are often evaluated independently of one another,^5^^,^^7^^,^^8^ which makes it difficult to identify which methods may be best suited for clinically important use cases. Addressing this gap in the literature in the context of extracting daily dosage from free-text sigs was the goal of this study.

In this study, we evaluated 5 algorithms identified during an initial scoping review of available methodologies for extracting daily dosage from free-text sigs. This includes the dosage a patient takes daily and the duration for which the patient needs to take this dosage. Methodologies were selected based on their documented or anticipated proficiency in daily-dose extraction.^5^^,^^6^^,^^9^

## Methods

### Data

A dataset of free-text sigs, specifically focusing on HFrEF, was collected from University of Utah Health, an academic healthcare system. The targeted medications were angiotensin-converting enzyme inhibitors, angiotensin receptor blockers, angiotensin receptor-neprilysin inhibitors, cardioselective beta blockers (bisoprolol, carvedilol, and metoprolol succinate), mineralocorticoid receptor antagonists, and sodium-glucose cotransporter-2 inhibitors. The medications were prescribed between January 1, 2023 and May 29, 2024, and the sigs originated from these prescriptions. One of the authors (T.S.H.) annotated the sigs manually to establish a gold standard. For any sigs with ambiguous or unclear meaning, a physician of internal medicine (J.S.C.) provided interpretation to facilitate annotation. Any sigs written in a language other than English were excluded. The dataset was de-identified and double-checked for any protected health information. Sigs for which it was impossible to extract a daily dosage were kept in the dataset to identify cases where a daily dosage should not be extracted. This means all algorithms had to accurately detect the absence of daily dosage information within a sig.

To fully capture all information presented in the sigs, the gold standard was represented using a data structure. This data structure is referred to as a “period object” for easy reference. Each period object contains information about a medication period for a sig from the dataset, as well as information about a specific dosage the patient should take, either for a designated duration or until a condition is met. In the dataset, sigs had up to 5 medication periods. The variables extracted for a medication period are displayed in Table 1. Only the variables highlighted in bold were included in the final evaluation, as they represent the daily dosage.

**Table 1.** Variables extracted from free-text sigs for each period object.

Variable	Description
Optional_Period	Indicates if the medication period is optional for the patient
Start_Condition	Starting condition for the medication period
**Daily_Dose_Low**	The lowest dose a patient may take
**Daily_Dose_High**	The highest dose a patient may take
**Daily_Dose_Units**	Units of the daily dose (eg, tab, cap, or pill)
**AltDaily_Dose_Low**	The lowest alternate dose a patient may take
**AltDaily_Dose_High**	The highest alternate dose a patient may take
**AltDaily_Dose_Units**	Units of the alternate daily dose (eg, mg or mL)
**Duration_Low**	The shortest amount of time for which the patient should take the dosage
**Duration_High**	The longest amount of time for which the patient should take the dosage
**Duration_Units**	Units of the duration (eg, day, week, or month)
Hold_Condition	The reason for the patient to not take the dosage

Variables used in the final evaluation are highlighted in bold.

The study was approved by the University of Utah Institutional Review Board (protocol 00173914). Explicit approval was granted for the use of an LLM.

### Algorithms evaluated

The following open-source algorithms were evaluated: Parsigs,^10^ RxSig (developed by one of the authors and available through the open-source OpenCDS repository),^11^ and Sig2db.^7^^,^^12^ Moreover, 2 machine learning approaches were evaluated: bidirectional long short-term memory (BiLSTM)^8^ and GPT4o,^13^ a LLM. During this study, the previously listed algorithms were used as a foundation, then adapted as necessary to enable extraction of daily dosages. Adaptations were made in accordance with each original algorithm as follows:

- If any input modifications to the sig text were necessary for the algorithm to work properly, they were adjusted (eg, numbers converted to words, eg, “2” to “two”).
- The input was split on the keywords “then” and “increase to” if this improved the algorithm’s extraction capabilities on the training set.
- The output of the algorithm was parsed into the standardized form (Table 1).

The adaptations, along with the training for the machine learning models, were conducted on a 70% random subset of the data, with the remaining 30% reserved for validation. The above adaptations were iteratively implemented for each of the sig parsing methodologies to ensure that the algorithms were appropriately configured for extracting daily dosage.

### RxSig

RxSig is an open-source algorithm designed by one of the authors (K.K.) to deterministically retrieve daily dosages from free-text sigs using regular expression patterns (eg, “((^q)|(q))([\s]?)([\d]+)((-)([\d]+))?([\s]?)((hr$)|(h$)|(hr)|(h)|(month)|(week)|(day)|($)|(prn))” for parsing intervals, such as “q4hr” into “every 4 hours” or “q2-3hr” into “every 2 to 3 hours”).^11^ It is important to note that RxSig was developed prior to this study, with a primary purpose of parsing opioid sigs, and thus was not influenced by any of the data used in this research. It first normalizes the sig text by ensuring all numbers are represented numerically, removing any text known to be irrelevant to daily-dose extraction, and parsing known synonyms into standardized forms (eg, “wk” to “week”).

From the normalized sig text, structured elements are then extracted. If any numbers other than dates remain in the leftover sig text, the algorithm tags the extracted sig data as uncertain. During the evaluation, sigs with uncertain sig data were categorized as unparsed by the algorithm.

### Sig2db

Sig2db is an algorithm that utilizes MetaMap, a tool for extracting UMLS concepts, to tag words within a clinical context.^14^ It then parses the output into a structured sig format. These structured sig elements are subsequently parsed to retrieve the daily dosage.^7^^,^^12^ Metamap 14 for Windows was used for this study.

### ParSigs

ParSigs is a named entity recognition model designed for speech tagging trained using SpaCy, a natural language processing library.^15^ It is specifically tailored to retrieve relevant information from sig text.^10^^,^^16^ The algorithm outputs structured variables that contain usable information for determining the daily dosage.

### BiLSTM

A BiLSTM model is a type of neural network that enables recurrent information processing.^8^ When a sentence is used as input, its words are sequentially fed into the model. Due to the model being bidirectional, word feeding occurs from both the start and the end of the sentence simultaneously. This approach allows for capturing more contextual information.

In preparation for using a BiLSTM model, all variables were converted to categorical variables. The categories were determined based on the distinct values each variable could hold. For example, the variable Daily_Dose_Units was divided into categories, such as Tablet, Pill, and Capsule, corresponding to the values present in Daily_Dose_Units. This methodology allowed for testing the capabilities of the BiLSTM model but hampers generalizability. Numerical variables could only fall within a certain range of numbers, always rounded to 0.25. Therefore, the numerical variables were better for training when split into categories, ranging from their lowest to their highest values. The model returned a percentage for each category in each variable specifying the chance of that category being the right one. The highest output percentage for a categorical variable was chosen as the output category for that variable.

Since neural networks expect numerical inputs, sentences need to be numerically vectorized. This was done using BERT-Large, a pre-trained contextual word-embedding model.^17^ BERT-Large was chosen because it has been found to be the best contextual embedding algorithm for some clinical contexts.^18^

The following hyperparameters were chosen during hyperparameter tuning using the specified training set, which was split into initial training and tuning sets of 80% and 20%, respectively, and tuned using random grid search. The final BiLSTM model was trained on both the training and tuning sets before testing on the separate test set. The hyperparameters used in the model were 1024 input layers, 2 hidden layers with 256 neurons, and a total of 317 output layers. It was trained using a learning rate of 0.001 and binary cross-entropy loss as its loss function.

### LLM

GPT-4o, an LLM from OpenAI, was selected due to its robust information extraction capabilities.^19^ The model was optimized by prompt engineering using few-shot information extraction techniques recommended by deeplearning.ai and OpenAI’s ChatGPT Prompt Engineering for Developers course.^20^ The final structured daily dosage information was extracted using a prompt depicted in Appendix S1 and parsed using ChatGPT’s Python application programming interface. GPT-4o was utilized in a way that ensured data was not used for model training.

### Outcome metrics

The daily dosage was considered to be correctly extracted if the extracted dosage the patient takes daily and the extracted duration for which the patient needs to take this dosage were both correct. Therefore, all variables of all medication periods needed to match those in the gold standard exactly. The variables Optional_Period, Start_Condition, and Hold_Condition were not considered in the analysis due to the scope of this study being daily dosage extraction. The unit of analysis was the sig, and we did not consider the possible correlation of sigs within a patient.

The outcome metrics used were positive predictive value (PPV), sensitivity, F1-score, run time, and costs. All variables except for run time and costs have a value range from zero to one. The following definitions were used:

- True positives (TP): cases where a daily dose was extracted, and correct.
- False positives (FP): cases where a daily dose was extracted, but incorrect.
- True negatives (TN): cases where no daily dose was extracted, when it was not possible to extract a daily dose.
- False negatives (FN): cases where a daily dose was not extracted but could have been extracted.
- PPV: calculated as TP/(TP + FP).
- Sensitivity: calculated as TP/(TP + FN).
- F1-score: calculated as 2 × (PPV × Sensitivity)/(PPV + Sensitivity).
- Time to finish: the time it took for the algorithm to return all the daily dosages of the test set in minutes.
- Cost to compute: the amount of money paid in US dollars to run the entire test set.

The PPV shows the proportion of correctly extracted daily dosages divided by all extracted daily dosages. Sensitivity shows how many sigs from which a daily dosage could have been extracted were correctly extracted. The F1-score is the harmonic mean of PPV and sensitivity (also called precision and recall, respectively, in the information retrieval literature). To evaluate run time, the algorithms were executed in the same environment, using a consistent method to parse the outcomes into a structured format. For time efficiency, only unique sigs were parsed to determine run time and costs. This does not affect the results otherwise.

To evaluate whether algorithm performance was consistent across subcategories of the dataset, subgroup analyses were conducted based on demographics, focusing on sex, race, and ethnicity, as well as on medication drug class. For the sex-based analysis, patients without a recorded sex in the electronic health record (EHR) were excluded. For the race and ethnicity analysis, patients were categorized as non-minority if their race was recorded as White and their ethnicity as non-Hispanic in the EHR. Patients were categorized as minority if their race was recorded as anything other than White (eg, Black or African American, American Indian or Alaska Native, Asian, Native Hawaiian or Other Pacific Islander, other, or multi-racial) or if their ethnicity was recorded as Hispanic. Patients with insufficient EHR data to determine minority status based on these definitions were excluded from the analysis. The following drug classes were present in the dataset and analyzed: angiotensin-converting enzyme inhibitor, cardioselective beta blocker, angiotensin receptor blocker, angiotensin receptor-neprilysin inhibitor (ARNI), sodium-glucose cotransporter-2 inhibitor, and mineralocorticoid receptor antagonist.

To test for statistically significant differences in performance on the test set, a permutation test was conducted on the best-scoring algorithms. This method provided further investigation into the most effective approach and its validity.

The environment used for training and testing was equipped with dual Intel^®^ Xeon^®^ Gold 6152 CPUs running at 2.10 GHz, with 16.0 GB of RAM, and operating on Windows 11.

## Results

A total of 29 896 free-text sigs from 17 295 patients were analyzed, from which 5 sigs were removed due to ambiguous meaning, and 56 sigs were removed for not being in English. The final set consisted of 29 835 sigs, of which 5633 were unique. These could be parsed into 604 different outcome patterns.

Table 2 reports the PPV, sensitivity, F1-score, time to finish, and cost to compute for the 5 algorithms on the test set. PPV ranged from 0.56 to 0.99, with the LLM and RxSig both scoring above 0.90. Sensitivity was high across all algorithms, consistently scoring above 0.90. The F1 score ranged from 0.71 to 0.98, with the LLM and RxSig scoring above 0.90. Time to finish varied between 4 (0.10 s per sig) and 210 min (5.30 s per sig), with RxSig and ParSigs completing in less than 10 min. The cost to compute was zero for all algorithms except for GPT4o, which cost 25 US dollars.

**Table 2.** Summary of outcome metrics for different daily dosage extraction algorithms.

Method	PPV	Sensitivity	F1-score	Time to finish (min)	Cost to compute (US$)	Time to finish/sig processed (s)
BiLSTM	0.56	0.99	0.71	34	0	0.85
LLM	0.96	1.00	0.98	210	25	5.30
Parsigs	0.84	0.93	0.88	9	0	0.23
RxSig	0.99	0.92	0.95	4	0	0.10
Sig2db	0.65	1.00	0.78	132	0	3.30

Abbreviations: BiLSTM: bidirectional long short-term memory; LLM: large language model.

Table 3 reports the PPV, sensitivity, and F1-score for the subcategories of sex (female/male) and race and ethnicity (minority/non-minority). Differences in performance metrics ranged from 0 to 0.03 points. The largest observed difference, 0.03 points, occurred in the BiLSTM male/female subgroups on PPV and in the RxSig female/male subgroups on sensitivity.

**Table 3.** Outcome metrics by demographic subgroup for different daily dosage extraction algorithms.

Method	Subgroup	PPV	Sensitivity	F1-score
BiLSTM	Female	0.59	0.99	0.74
	Male	0.62	0.99	0.76
	Minority	0.62	1.00	0.76
	Non-minority	0.60	0.99	0.75
LLM	Female	0.97	1.00	0.98
	Male	0.98	1.00	0.99
	Minority	0.97	1.00	0.98
	Non-minority	0.98	1.00	0.99
Parsigs	Female	0.87	0.95	0.90
	Male	0.88	0.95	0.91
	Minority	0.87	0.95	0.91
	Non-minority	0.87	0.95	0.91
RxSig	Female	0.99	0.92	0.96
	Male	0.99	0.95	0.97
	Minority	0.99	0.93	0.96
	Non-minority	0.99	0.94	0.96
Sig2db	Female	0.69	1.00	0.82
	Male	0.71	1.00	0.83
	Minority	0.70	1.00	0.83
	Non-minority	0.70	1.00	0.82

Abbreviations: BiLSTM: bidirectional long short-term memory; LLM: large language model.

Table 4 reports the PPV, sensitivity, and F1-score for the medication drug class subcategories. The LLM and RxSig methods showed the least variation between medication drug class subcategories, with a maximum difference of 0.02. For the beta blocker drug class, the PPV in the BiLSTM, ParSIGs, and Sig2DB methods was 0.2 to 0.27 points lower than their highest PPV score. The lowest F1 score was observed for the ARNI drug class in the BiLSTM method.

**Table 4.** Outcome metrics by drug class subgroup for different daily dosage extraction algorithms.

Method	Subgroup	PPV	Sensitivity	F1-score
BiLSTM	ACE inhibitor	0.68	1.00	0.81
	Beta blocker	0.41	0.98	0.57
	ARB	0.66	0.99	0.79
	ARNI	0.27	1.00	0.43
	SGLT-2 inhibitor	0.65	1.00	0.78
	MRA	0.44	0.98	0.61
LLM	ACE inhibitor	0.98	1.00	0.99
	Beta blocker	0.96	1.00	0.98
	ARB	0.98	1.00	0.99
	ARNI	0.99	1.00	1.0
	SGLT-2 inhibitor	0.99	1.00	0.99
	MRA	0.94	1.00	0.97
Parsigs	ACE inhibitor	0.75	1.00	0.86
	Beta blocker	0.55	1.00	0.71
	ARB	0.75	1.00	0.85
	ARNI	0.74	1.00	0.85
	SGLT-2 inhibitor	0.75	1.00	8.6
	MRA	0.59	1.00	0.75
RxSig	ACE inhibitor	0.99	0.93	0.96
	Beta blocker	0.99	0.93	0.96
	ARB	1.00	0.94	0.97
	ARNI	1.00	0.96	0.98
	SGLT-2 inhibitor	0.99	0.95	0.97
	MRA	0.98	0.88	0.93
Sig2db	ACE inhibitor	0.75	1.0	0.86
	Beta blocker	0.55	1.0	0.71
	ARB	0.75	1.0	0.85
	ARNI	0.74	1.0	0.85
	SGLT-2 inhibitor	0.75	1.0	0.86
	MRA	0.59	1.0	0.75

Abbreviations: ACE: angiotensin-converting enzyme; ARB: angiotensin receptor blocker; ARNI: angiotensin receptor-neprilysin inhibitor; MRA: mineralocorticoid receptor antagonist; SGLT-2: sodium-glucose cotransporter-2.

Both the LLM and RxSig achieved the highest scores on daily-dose extraction, with F1-scores of 0.98 and 0.95, respectively. The difference in F1-score was primarily due to sensitivity, where RxSig scored 0.92 compared to 1.00 for the LLM. However, RxSig outperformed the LLM on PPV, scoring 0.99 compared to the LLM’s 0.96. This corresponds to the LLM correctly extracting 7051 sigs, compared to 6845 for RxSig. The LLM had 179 incorrect extractions, while RxSig had 71. However, despite differences in individual metrics, there was no statistically significant difference in the overall test set performance of RxSig and the LLM (*P* = 1.000). The LLM incurred a cost of $25 and had a time to finish of 210 min, which translates to 5.30 s per sig, whereas RxSig had no costs and took 4 min (0.10 s per sig) to finish. The BiLSTM model scored lowest, with an F1 score of 0.71, primarily due to its low PPV of 0.56.

## Discussion

### Key findings

The analysis revealed that both the LLM and RxSig achieved the highest scores on daily-dose extraction and that the BiLSTM model scored the lowest. The low PPV of BiLSTM indicated that its model correctly extracted only half of the sigs. In practical usage for clinical care, it would be preferable for an algorithm to score high in PPV, meaning almost all its extracted daily dosages are correct, while maintaining a good F1-score. For example, the LLM extracted the sig “take 1/2 tablet (50 mg) by mouth every day” as a dose of 25 mg per day, which is half of the actual prescribed dose. Mistakes in extracted daily dosages like this could lead to physicians relying on incorrect data to make decisions, whereas uncertainty identified by the parsing algorithm, indicating that it is unable to confidently make a prediction, is more likely to be accommodated by clinicians manually reviewing the sig. Additionally, long times to finish and high costs to compute are not scalable for larger datasets, making them potentially less practical for clinical use by health systems.^21^^,^^22^ RxSig scored consistently well in all these key clinical and operational outcome metrics.

No notable differences in performance metrics were observed between subgroups of sex (female/male) or race and ethnicity (minority/non-minority) for any of the evaluated algorithms, suggesting consistent algorithm performance across these demographic categories. Similarly, the RxSig and LLM methods showed minimal variation across medication drug classes, while other methods, such as the BiLSTM model, Parsigs, and Sig2DB, showed notable differences, particularly for beta blockers and ARNI, which scored lower. This likely reflects the greater complexity involved in extracting information for these drug classes.

### Implications

The LLM’s high score in daily-dose extraction is consistent with prior studies that demonstrated effectiveness of LLMs in information retrieval tasks across various settings.^19^ Although no prior studies had been conducted specifically on RxSig, its high score here demonstrates regular expressions’ utility in information extraction, as previously noted in the literature.^4^ However, the low score of the BiLSTM model contradicts existing literature, which suggested that BiLSTM shows promise for information extraction in the clinical context.^11^ This discrepancy may be attributable to the limited output variability in the dataset (eg, “1 tablet” or “2 tablets” for dose), which could have incentivized the model to return a limited number of categories in order to minimize its loss function. Another contributor to this limited performance may have been the use of a computer with moderate computing power as opposed to a high-performance computing server. With more technical resources, the hyperparameter tuning could have been conducted more extensively, leading to an improved model. Additionally, this would have enabled the usage of *k*-fold cross-validation.

This study adds to an increasing body of literature that has found that general-purpose LLMs such as GPT-4 perform very well in the extraction of desired information from free text with limited effort, but that they suffer from hallucinations (false-positive findings in this case).^6^^,^^23^ Furthermore, such LLMs may not perform as well as expert-curated approaches (eg, RxSig) with regard to key metrics of interest for operational clinical use (eg, PPV, execution speed, cost to compute).^24^ Thus, while very useful and promising, LLMs should be used with caution in tasks such as the extraction of daily dosage from free-text sigs.

### Strengths and limitations

One strength of this study is the comparison of multiple sig-parsing algorithms, including LLMs. Second, all algorithms were compared for clinically and operationally relevant metrics, ensuring their applicability to clinical use. Third, this study used actual clinical data for a clinically relevant use case. A limitation of this study is its reliance on a single health system and a single targeted condition, which may limit the generalizability of its findings when extracting sigs in diverse or different healthcare systems. Additionally, limited computational resources could have resulted in suboptimal hyperparameter tuning and predictive performance of the BiLSTM model. These constraints also prevented the use of *k*-fold cross-validation, a key approach to avoid overfitting. Finally, annotation of the dataset was completed before the algorithms were adapted. This could have introduced potential bias, as the sigs in the validation set may have already been seen by the researcher conducting the adaptations (T.S.H.). However, given the thousands of unique sigs, such bias due to recall is unlikely.

### Future research

Further research is needed to investigate the generalizability of the findings across diverse health systems and target conditions. The BiLSTM approach could also be further evaluated using larger data sets and more powerful computational resources, also enabling the use of *k*-fold cross-validation. Additionally, this would allow for a more extensive analysis across a wider range of subcategories.

Additionally, when spans in the sig are flagged, methods to learn regular expressions from data could be explored to enhance deterministic approaches like RxSig. For LLMs, prompt engineering techniques could be employed to instruct models to explicitly indicate uncertainty in their outputs. This functionality would improve the efficiency and safety of clinical workflows by focusing attention on cases where the algorithms know they are less certain. Other types of models may benefit from considering an out-of-distribution score to accompany their predictions. The higher the score, the less confident the model is in its prediction.

Also of interest would be identifying whether and how LLMs should be incorporated into this information extraction task. For example, a deterministic approach such as RxSig could be used as a first-line approach, supplemented with the use of LLMs in cases where RxSig is unable to extract the data with confidence. In such cases, the LLM’s output could be flagged for human review. Another possible approach may be to use multiple approaches (eg, RxSig and GPT-4o), and consider the results to be of high confidence if they both agree. Furthermore, the results of relatively slow and expensive approaches such as GPT-4o could be cached for a distinct input pattern to speed up execution speed and minimize costs.

Another consideration is the potential utility of the extracted sig data for patient-facing communication, such as through the Universal Medication Schedule (UMS).^25^ The outputs of the models could be adapted to provide standardized, patient-friendly instructions in line with UMS principles. This would enable the models to be used in a more patient-centric approach, enhancing understanding and supporting improved medication adherence.^26^

## Conclusion

This study demonstrates that both the LLM and RxSig models excelled in the extraction of daily doses from free-text sigs for heart failure prescriptions. RxSig stood out as a particularly scalable approach, offering high PPV, low costs, and short running times. Future research is warranted to evaluate generalizability and the appropriate role of LLMs in the extraction of daily doses from free-test sigs.

## Supplementary Material

ooae153_Supplementary_Data

## Data Availability

The data underlying this article will be shared on reasonable request to the corresponding author.
