# Supplementary material for: Approaches for extracting daily dosage from free-text prescription signatures in heart failure with reduced ejection fraction: a comparative study
Source: JAMIA Open. 2025 Jan 3;8(1):ooae153. doi: 10.1093/jamiaopen/ooae153 (PMC11700559; doi:10.1093/jamiaopen/ooae153)
Supplement: ooae153_Supplementary_Data [file ooae153_supplementary_data.docx]

## Appendix 1

The below prompt was sent to the Python ChatGPT application programming interface (API) to retrieve the daily dosage of a sig. The response was captured in JavaScript Object Notation (JSON) format [25]. The prompt variable was sent as a “prompt” attribute, the response, comp1, and comp2 variables were combined into an “assistant” attribute, and the user1 and user2 were sent as a “user” attribute. These attributes were consecutively fed into the API.

prompt = (

        "From the following free-text prescription signature that I will send I want you to retrieve the daily dose."

        "Return a Jason with every by brackets delimited sig. The daily dosage needs to be given in a structured json format. "

        "The format consists of 5 medication periods. A medication period contains the following keys {key} and information {info}: \n\n"

        "DailyDoseLow{key} the lowest daily dose of a medication the patient may take{info} \n"

        "DailyDoseHigh{key} the highest daily dose of a medication the patient may take{info} \n"

        "DailyDoseUnit{key} the unit of the daily dose being tablet, pill or capsule{info}\n"

        "AltDailyDoseLow{key} the lowest alternate daily dose of a medication the patient may take{info} \n"

        "AltDailyDoseHigh{key} the highest alternate daily dose of a medication the patient may take{info} \n"

        "AltDailyDoseUnit{key} the unit of the daily dose being milligrams (mg) or milliliters (ml){info} \n"

        "DurationLow{key} lowest amount of time the patient needs to take the medication in this period{info}\n"

        "DurationHigh{key} longest amount of time the patient needs to take the medication in this period{info}\n"

        "DurationUnit{key} unit of the amount of time the patient needs to take the medication being day, week or month\n\n"

        "A medication period is filled when necessary. Only give the medication periods that are not empty."

        "A medication period ends or starts when the sig describes a change in the amount to be taken. "

        "When with is present in the sig only report back on the type of dosage that is named twice. Examples:\n\n"

        "input: [take one tablet a day] \n"

        "output: {“sig”:”take one tablet a day”,”periods”:[{“DailyDoseLow”:1,“DailyDoseHigh”:1, “DailyDoseUnit”:”tablet”,“AltDailyDoseLow”:None,“AltDailyDoseHigh”:None, “AltDailyDoseUnit”:None, “DurationLow”:None, “DurationHigh”:None,”DurationUnit”:None}]}\n\n"

        "input: [take one pill 10mg every pm and two pills 20mg every am for 5 days] \n"

        "output: {“sig”:”take one pill 10mg every pm and two pills 20mg every am for 5 days”,”periods”:[{“DailyDoseLow”:3,“DailyDoseHigh”:3, “DailyDoseUnit”:”pill”,“AltDailyDoseLow”:30,“AltDailyDoseHigh”:30, “AltDailyDoseUnit”:”mg”, “DurationLow”:5, “DurationHigh”:5,”DurationUnit”:”day”}]}\n\n"

        "input: [take one up to two tabs every day. Strength: 50 mg] \n"

        "output: {“sig”:”take one up to two tabs every day. Strength: 50 mg”,”periods”:[{“DailyDoseLow”:1,“DailyDoseHigh”:2, “DailyDoseUnit”:”tablet”,“AltDailyDoseLow”:50,“AltDailyDoseHigh”:100, “AltDailyDoseUnit”:”mg”, “DurationLow”:None, “DurationHigh”:None,”DurationUnit”:None}]}\n\n"

        "input: [take two tabs 20 mg every day] \n"

        "output: {“sig”:”take two tabs 20 mg every day”,”periods”:[{“DailyDoseLow”:2,“DailyDoseHigh”:2, “DailyDoseUnit”:”tablet”,“AltDailyDoseLow”:20,“AltDailyDoseHigh”:20, “AltDailyDoseUnit”:”mg”, “DurationLow”:None, “DurationHigh”:None,”DurationUnit”:None}]}\n\n"

        "input: [take two tabs 40 mg twice a day] \n"

        "output: {“sig”:”take two tabs 20 mg every day”,”periods”:[{“DailyDoseLow”:4,“DailyDoseHigh”:4, “DailyDoseUnit”:”tablet”,“AltDailyDoseLow”:80,“AltDailyDoseHigh”:80, “AltDailyDoseUnit”:”mg”, “DurationLow”:None, “DurationHigh”:None,”DurationUnit”:None}]}\n\n"

        "input: [take one pill (100mg) every monday, wednesday and friday for 10 up to 12 days then take 1 pill (100mg) every other day] \n"

        "output: {“sig”:”take one pill (100mg) every monday, wednesday and friday for 10 days then take 1 pill (100mg) every other day”,”periods”:[{“DailyDoseLow”:0.43,“DailyDoseHigh”:0.43, “DailyDoseUnit”:”pill”,“AltDailyDoseLow”:42.9,“AltDailyDoseHigh”:42.9, “AltDailyDoseUnit”:”mg”, “DurationLow”:10, “DurationHigh”:12,”DurationUnit”:”day”},{“DailyDoseLow”:0.5,“DailyDoseHigh”:0.5, “DailyDoseUnit”:”pill”,“AltDailyDoseLow”:50,“AltDailyDoseHigh”:50, “AltDailyDoseUnit”:”mg”, “DurationLow”:None, “DurationHigh”:None,”DurationUnit”:None}]}\n\n"

        "input: [take one tablet 20 mg a day with 40 mg] \n"

        "output: {“sig”:”take one tablet 20 mg a day with 40 mg”,”periods”:[{“DailyDoseLow”:None,“DailyDoseHigh”:None, “DailyDoseUnit”:”tablet”,“AltDailyDoseLow”:60,“AltDailyDoseHigh”:60, “AltDailyDoseUnit”:mg, “DurationLow”:None, “DurationHigh”:None,”DurationUnit”:None}]}\n\n"

        "input: [take one tablet 10 mg twice a day with 40 mg for a total of 50 mg twice a day] \n"

        "output: {“sig”:”take one tablet 10 mg twice a day with 40 mg for a total of 50 mg twice a day”,”periods”:[{“DailyDoseLow”:None,“DailyDoseHigh”:None, “DailyDoseUnit”:”tablet”,“AltDailyDoseLow”:100,“AltDailyDoseHigh”:100, “AltDailyDoseUnit”:mg, “DurationLow”:None, “DurationHigh”:None,”DurationUnit”:None}]}\n\n"

        "input: [take two tablets 20 mg a day] \n"

        "output: {“sig”:”take two tablets 20 mg a day”,”periods”:[{“DailyDoseLow”:2,“DailyDoseHigh”:2, “DailyDoseUnit”:”tablet”,“AltDailyDoseLow”:20,“AltDailyDoseHigh”:20, “AltDailyDoseUnit”:mg, “DurationLow”:None, “DurationHigh”:None,”DurationUnit”:None}]}\n\n"

        "input: [take three tablet 40 mg a day] \n"

        "output: {“sig”:”take three tablet 40 mg a day”,”periods”:[{“DailyDoseLow”:3,“DailyDoseHigh”:3, “DailyDoseUnit”:”tablet”,“AltDailyDoseLow”:40,“AltDailyDoseHigh”:40, “AltDailyDoseUnit”:mg, “DurationLow”:None, “DurationHigh”:None,”DurationUnit”:None}]}\n\n"

    )

    response = ("Please provide the free-text prescription signature delimited by square brackets, and I will process it to retrieve the daily dose in the specified JSON format.")

    user1 = ("Pay attention: when present in a sig X tablets Y mg than Y divided by X is the amount of mg per tablet. [take three tablets 20 mg twice a day]")

    comp1 = ("{“sig“: “take three tablets 20 mg twice a day“,“periods“: [{“DailyDoseLow“: 6,“DailyDoseHigh“: 6,“DailyDoseUnit“: “tablet“,“AltDailyDoseLow“: 120,“AltDailyDoseHigh“: 120,“AltDailyDoseUnit“: “mg“,“DurationLow“: null,“DurationHigh“: null,“DurationUnit“: null}]}")

    user2 = ("Pay attention")

    comp2 = ("Here's the corrected output, considering the correct calculation for the dosage in mg per tablet: {“sig“: “take three tablets 20 mg twice a day“,“periods“: [{“DailyDoseLow“: 6,“DailyDoseHigh“: 6,“DailyDoseUnit“: “tablet“,“AltDailyDoseLow“: 40,“AltDailyDoseHigh“: 40,“AltDailyDoseUnit“: “mg“,“DurationLow“: null,“DurationHigh“: null,“DurationUnit“: null}]}")
